# Risk factors predicting osteosarcopenia in postmenopausal women with osteoporosis: A retrospective study

**DOI:** 10.1371/journal.pone.0237454

**Published:** 2020-08-07

**Authors:** Hiroki Okamura, Koji Ishikawa, Yoshifumi Kudo, Akira Matsuoka, Hiroshi Maruyama, Haruka Emori, Ryo Yamamura, Chikara Hayakawa, Soji Tani, Koki Tsuchiya, Toshiyuki Shirahata, Tomoaki Toyone, Takashi Nagai, Katsunori Inagaki

**Affiliations:** Department of Orthopaedic Surgery, Showa University School of Medicine, Tokyo, Japan; Ehime University Graduate School of Medicine, JAPAN

## Abstract

There is growing interest in “osteosarcopenia” as the coexistence of osteoporosis and sarcopenia exacerbates negative outcomes. However, limited information is available regarding the risk factors of osteosarcopenia development in patients with osteoporosis. Therefore, we retrospectively reviewed 276 consecutive patients with postmenopausal osteoporosis who regularly visited Showa University Hospital. Patients were eligible for the study if they were ≥65 years of age and underwent dual-energy X-ray absorptiometry, blood sampling, and physical performance assessment. Patients were divided into the osteosarcopenia and osteoporosis alone groups according to the diagnostic criteria of the Asian Working Group for Sarcopenia. Of the 276 patients with osteoporosis, 54 patients (19.6%) had osteosarcopenia. Patients in the osteosarcopenia group had a greater risk of frailty than did those in the osteoporosis alone group (odds ratio 2.33; 95% confidence interval, 1.13–4.80, P = 0.028). Low body mass index seemed to be the strongest factor related to the development of osteosarcopenia, and none of the patients in the osteosarcopenia group were obese (BMI ≥27.5 kg/m^2^). Multiple logistic analyses revealed that patients aged 65–74 years who had comorbidities such as kidney dysfunction and high levels of HbA1c were at risk of developing osteosarcopenia. Thus, we strongly recommend the assessment of the key components of the diagnosis of osteosarcopenia in an osteoporosis clinic for patients with low body mass index. Furthermore, appropriate assessments, including comorbidities, will help in identifying patients at greater risk of developing osteosarcopenia.

## Introduction

Although the aging population is rapidly increasing worldwide, Japan is the foremost super-aging society, with no parallel in history. As the world’s population is aging, the prevalence of chronic diseases has increased. Hence, research on various countermeasures against musculoskeletal diseases, which impair daily living and the quality of life, is urgently needed [1–3].

Osteoporosis and sarcopenia, two major musculoskeletal diseases, are different conditions but share pathophysiological pathways associated with aging [4]. Genetic, endocrine, and mechanical factors have effects on both bone and muscle [4–7]. The common mesenchymal origin of bone and muscle cells might underpin the tight link seen between osteoporosis and sarcopenia [4]. Osteoporosis is one of the most common public health issues and is a powerful risk factor for adverse health outcomes, such as fractures [8]. Sarcopenia, which reflects age-related loss in skeletal muscle mass, strength, and function, is characterized by the deterioration of muscle quantity and quality, often leading to severe adverse outcomes [9, 10]. As the coexistence of osteoporosis and sarcopenia exacerbates negative health outcomes and has been described as a “hazardous duet,” there is a growing interest in osteoporosis and sarcopenia, resulting in the creation of the terms “osteo-sarcopenia” and “sarco-osteoporosis” [9–13]. Furthermore, a recent population-based cohort study reported that a large population of patients with sarcopenia had osteoporosis, and the presence of osteoporosis significantly increased the risk of osteosarcopenia development [11, 14].

The prevention of age-related changes, which are universal human phenomena, is unlikely to be approved as a treatment. However, a recent systematic review and meta-analysis showed the existence of some positive effects of exercise and nutrition interventions for the treatment of sarcopenia in older people [15]. Various factors are believed to be involved in osteosarcopenia. However, the risk factors of osteosarcopenia development in patients with osteoporosis remain unknown. Therefore, there is a clinical need to better understand which patients, especially patients with osteoporosis, are at risk of developing osteosarcopenia. The aim of the present study was to understand the clinical, functional, and biochemical features of patients with osteoporosis who are at risk of developing osteosarcopenia. The results provide new insight regarding the possible risk factors of disease progression from osteoporosis to osteosarcopenia.

## Materials and methods

### Study population

In this retrospective cross-sectional study, the medical records of 609 patients with osteoporosis, who regularly visited Showa University Hospital in Tokyo, Japan, were reviewed. Osteoporosis was defined in accordance with the Japan Osteoporosis Society’s diagnostic criteria for primary osteoporosis (JOS criteria) [16]. At our center for osteoporosis, patients annually undergo dual-energy X-ray absorptiometry (DXA), blood sampling, and physical performance assessment.

Patients were eligible for the study if they met the following criteria: (1) age ≥ 65 years, (2) diagnosed of postmenopausal osteoporosis, and (3) blood sampling and DXA performed on the same day between October 1, 2016, and September 31, 2017. On the basis of the initial criteria, 454 consecutive patients were eligible for study participation. Patients with osteoporosis other than postmenopausal osteoporosis (i.e., osteoporosis caused by malignant tumors, steroids, hyperthyroidism, and metabolic diseases) were excluded. We also excluded patients with severe chronic kidney disease who required hemodialysis. Additionally, 97 patients with incomplete data (missing physical performance measurements, DXA measurements, laboratory data, fall risk score, or Fried frailty score) were excluded. Finally, 276 patients (age: mean ± standard deviation (SD), 77.07 ± 6.74 years; range, 65–97 years) were included in the analyses.

**Fig 1:**
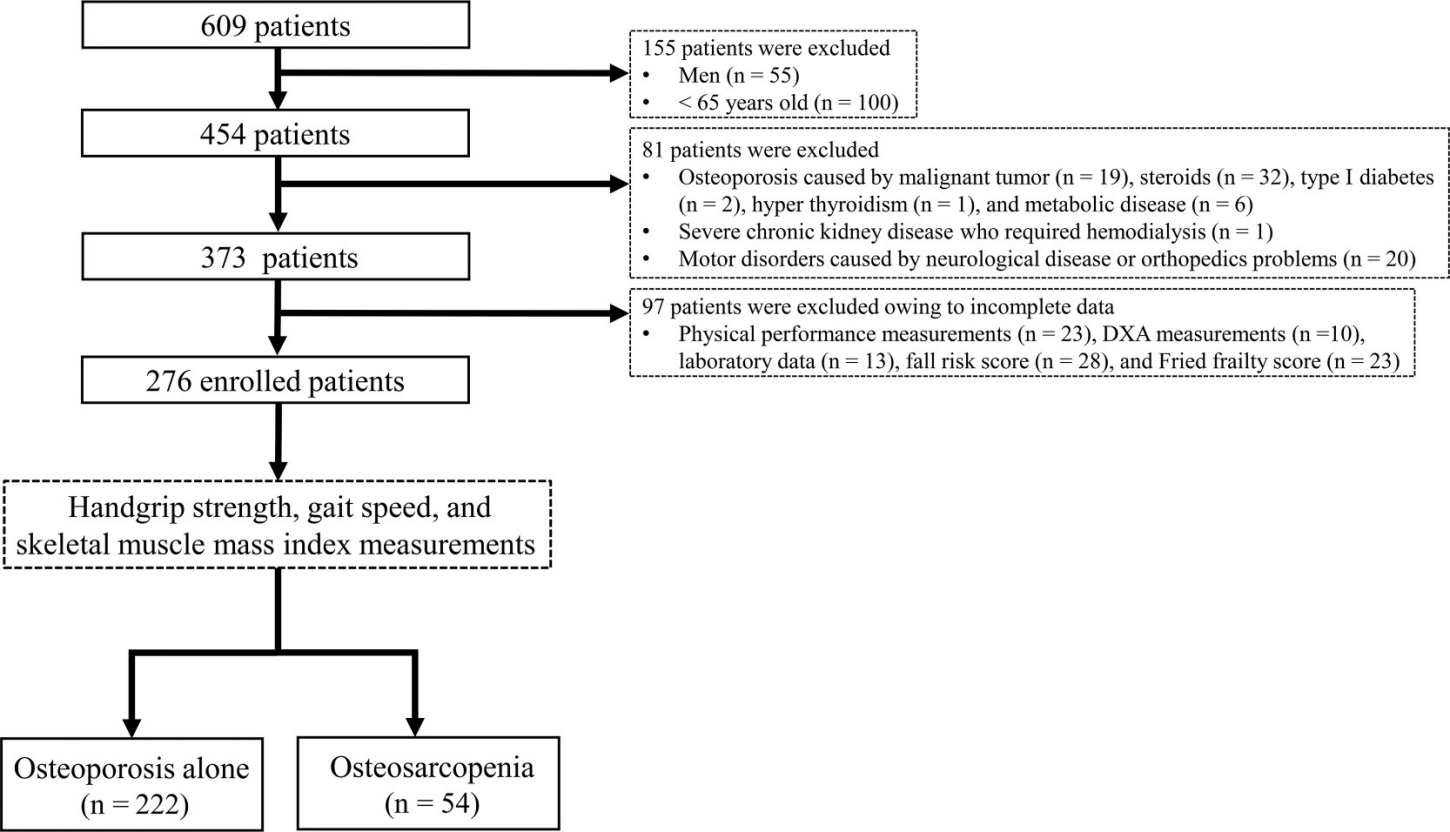
Flow diagram depicting the study’s patient selection process. DXA, dual-energy X-ray absorptiometry.

Patients were divided into the osteoporosis alone group (OP) and osteosarcopenia group (OS) according to the diagnostic criteria of the Asian Working Group for Sarcopenia (AWGS) [17]. OP comprised 222 (80.4%) patients, and OS comprised 54 (19.6%) patients. Fig 1 shows the flow diagram depicting the study’s patient selection process.

This study adhered to the tenets of the Declaration of Helsinki and was approved by the ethics committee of Showa University School of Medicine (No. 2699). Informed consent was waived because of the retrospective study using medical records.

### Data collection

We collected data on factors that may contribute to the conditions of sarcopenia and osteoporosis as follows: (1) age, (2) body mass index (BMI), (3) laboratory data, (4) previous osteoporosis fracture, (5) fall risk score, (6) Fried frailty score, (7) DXA measurements, (8) physical performance assessment, and (9) osteoporosis treatment. BMI was calculated as weight in kilograms divided by height in meters squared (kg/m^2^). BMI categories were defined by WHO recommendations for Asian populations as follows: [Emaciation: < 18.5 kg/m^2^, Normal weight: 18.5–22.9 kg/m^2^, Overweight: 23.0–27.4 kg/m^2^, Obesity: ≥27.5 kg/m^2^] [18]. The fall risk score was obtained using a validated questionnaire that was constructed in a prospective study with a large sample of participants who were older than 65 years [19].

#### Biochemical measurements

In all participants, blood and urine samples were obtained in a fasting state. Serum levels of albumin (Alb), alkaline phosphatase (ALP), calcium (Ca), phosphorus (P), eGFR, hemoglobin A1c (HbA1c), 25-hydroxy vitamin D (25-(OH) D), and insulin-like growth factor 1 (IGF-1) were assessed. Serum levels of intact parathyroid hormone (PTH) were obtained using an immunoradiometric assay kit (Elecsys PTH assay; Roche Diagnostics Inc., Tokyo, Japan; reference range [RR] in postmenopausal women: 10–65 pg/mL). In addition, bone turnover markers were assessed: bone-specific alkaline phosphatase (BAP) was estimated using Access Ostase (Beckman Coulter Inc., Tokyo, Japan; RR in postmenopausal women: 3.8–22.6 μg/L), and urine levels of crosslinked N-telopeptide of type I collagen (urinary-NTX; RR in postmenopausal women: 14.3–89.0 nmol bone collagen equivalent [BCE]/mmol creatinine [CRE]) were estimated using Osteomark (Abbott Inc., Tokyo, Japan).

#### Bone mineral density and skeletal muscle mass index as assessed by dual-energy X-ray absorptiometry

The bone mineral densities (BMDs) of the lumbar spine (LS-BMD; L2–4), femoral neck (FN-BMD), and distal radius (RUD-BMD) were measured using DXA (Hologic QDR series; Hologic, Waltham, MA). All DXA measurements were determined by a radiologist at a central site. The skeletal muscle mass index (SMI) was assessed by DXA on the basis of the theory that the fat-free mass (FFM) of the four limbs is approximately equal to the muscle mass (FFM/height [m^2^]) [20].

#### Physical performance measurements

Muscle strength was assessed as the handgrip strength, measured using a Smedley hand dynamometer (MY-2080; Matsumiya Ikaseiki Seisakusho Co., Ltd., Tokyo, Japan). One trial was performed for each hand, and the result from the stronger hand was used for the diagnosis of sarcopenia. Grip strength was measured three times, and the median was used in the analyses as described in the previous studies [21, 22]. We also assessed the usual gait speed, defined as the time taken to walk 4 m where ≤ 0.8 m/s suggests an increased risk of sarcopenia [17, 23]. To maintain the measurement of walking speed, patients were instructed to walk at their preferred speed over a length of 4 m, without slowing down before passing the 4 m line.

#### Fall risk score

In the present study, we used a simple screening questionnaire to determine the risk of falls in elderly patients [19]. The questionnaire items were as follows: (1) Do you have a history of falls within one year? (yes, 5 points); (2) Do you feel that your walking speed declined recently? (yes, 2 points); (3) Do you use a cane when you walk? (yes, 2 points); (4) Is your back bent? (yes, 2 points); and (5) Do you take more than five kinds of prescribed medicines? (yes, 2 points). (S1 Table) The fall risk score ranged from 0 to 13 points. In this screening test, the cut-off value is > 6 points [Odds ratio of 3.88 (95% CI: 3.16–3.88)].

#### Diagnosis of osteoporosis

All the patients in the present study met the JOS-criteria [24]. Briefly, osteoporosis is diagnosed by the bone mineral density (BMD) criteria or the occurrence of a fragility fracture as follows. [1. BMD is equal to or below either 70% or -2.5 standard deviations of young adult mean (YAM). 2. Presence of a fragility fracture in either the lumbar spine or the proximal femur. 3. Presence of another fragility fracture and a BMD below 80% of YAM.]

#### Diagnosis of sarcopenia

The diagnostic criteria of the AWGS, widely used in Asia since they were first published, were applied for the diagnosis of sarcopenia [17]. There are three components of factors: (1) low muscle mass (SMI < 5.4 kg/m^2^), (2) low muscle strength (grip strength < 18 kg), and (3) low physical function (walking speed ≤ 0.8 m/s). Thereby, the diagnosis of sarcopenia was based on the documentation of low muscle mass (SMI < 5.4 kg/m^2^) and [low muscle strength (grip strength < 18 kg) or low physical performance (walking speed ≤ 0.8 m/s)].

#### Diagnosis of frailty

The diagnosis of frailty was determined using the Fried frailty score, which is based on five physical indicators: (1) exhaustion, (2) low physical activity, (3) weakness, (4) slow walking, and (5) unintentional weight loss [22]. Each indicator was scored as either 0 or 1. Therefore, the total score ranged from 0 to 5 points. Patients were categorized as non-frail, pre-frail, or frail using established cut-off points of the total score (0, 1–2, and ≥3 points, respectively).

#### Diagnosis of osteo-sarcopenia, osteo-frailty, osteo-sarco-frailty

The definition of osteoporosis, sarcopenia, and frailty may overlap, because each diagnosis is a common negative consequence of aging. To elucidate how each diagnosis contributes to the overall structure of this population, further categories using JOS-criteria, AWGS, and Fried criteria were defined in the present study [17, 22, 24]. In accordance with these criteria osteo-sarcopenia was defined as the patients who met both JOS-criteria and AWGS. In the same manner, osteo-frailty and osteo-sarco-frailty were defined in patients who met following criteria: osteo-frailty; JOS criteria and Fried criteria, osteo-sarco-frailty; JOS criteria, AWGS, and Fried criteria.

#### Statistical analysis

Continuous variables with normal distribution are presented as mean ± standard deviation (SD), whereas nonnormally distributed variables are presented as median with interquartile range (IQR). Differences in background factors between OP and OS were evaluated using the Mann–Whitney *U* test or the chi-squared test, as appropriate. Clinical parameters were compared between OP and OS, overall and according to three age categories (65–74, 75–84, and ≥85 years). Univariate and multivariate logistic analyses were performed to identify factors affecting the presence of sarcopenia. Variables with a P-value < 0.15 on univariate logistic analysis except the components of sarcopenia diagnosis (handgrip strength, gait speed, and SMI) were submitted to multivariate logistic models using the stepwise method. All statistical analyses were performed using StatFlex version 6 (Artech Co., Ltd.). All statistical tests were two-tailed, and P-values < 0.05 were considered statistically significant.

## Results

### Baseline patient characteristics

Two hundred and seventy-six patients with postmenopausal osteoporosis were included in this study, with 80.4% (222/276) of the patients in OP and 19.6% (54/276) of the patients in OS. The mean age was 77.07 years, and the median BMI was 21.90 kg/m^2^. The median LS-BMD, FN-BMD, and RUD-BMD was 0.78 g/cm^2^, 0.51 g/cm^2^, and 0.27 g/cm^2^, respectively.

Among the 276 patients, 259 (93.8%) patients had received treatment for osteoporosis, which included bisphosphonate, selective estrogen receptor modulators, denosumab, teriparatide, and activated vitamin D. Seventeen patients were not under any medical osteoporosis treatment.

### Relationships among osteoporosis, sarcopenia, and frailty

**Fig 2:**
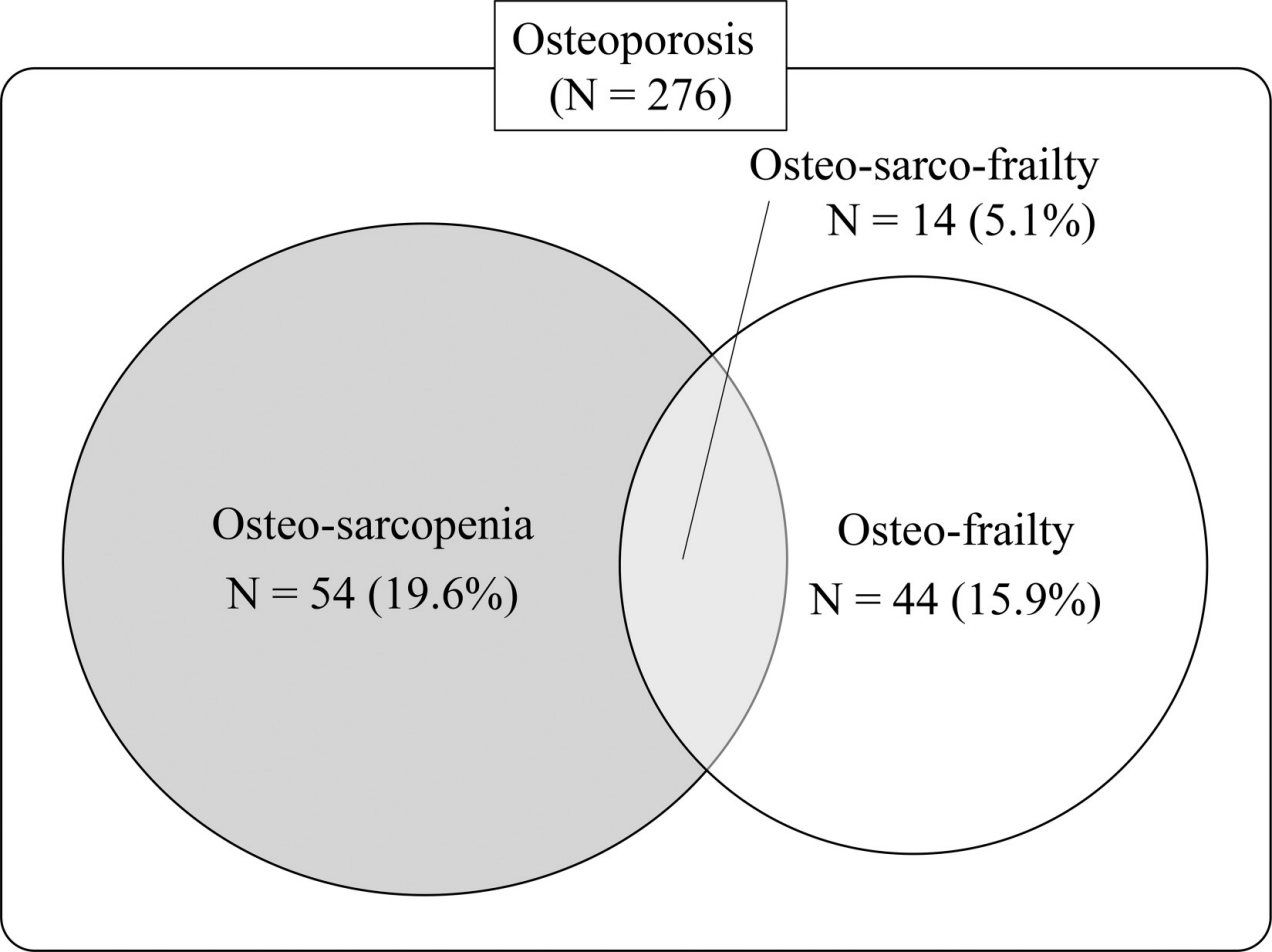
Venn diagram showing the overlap in osteoporosis, sarcopenia, and frailty. Osteo-sarcopenia refers to patients with osteoporosis and sarcopenia. Osteo-frailty refers to patients with osteoporosis and frailty. Osteo-sarco-frailty refers to patients with osteoporosis, sarcopenia, and frailty.

**Fig 3:**
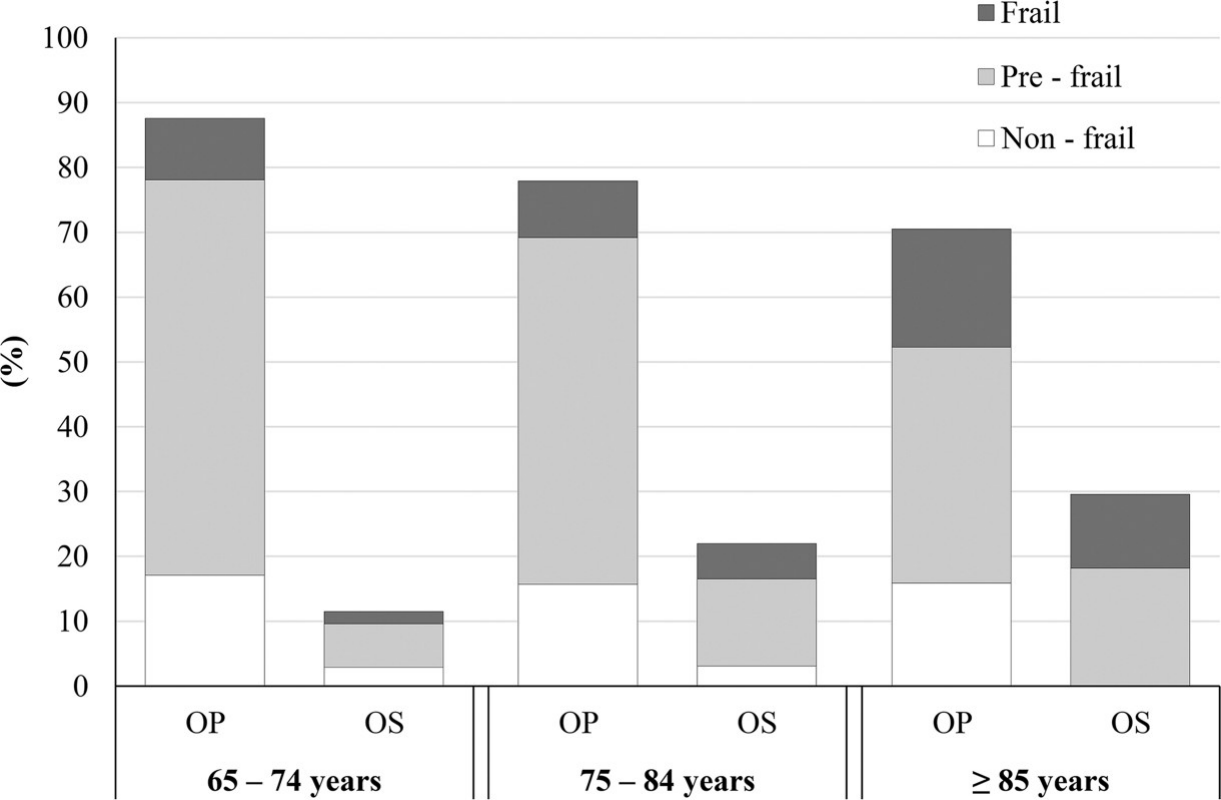
Prevalence of frailty in patients with osteoporosis alone and osteosarcopenia. OP, osteoporosis alone group; OS, osteosarcopenia group.

Fig 2 shows the prevalence of osteo-sarcopenia (patients with osteoporosis and sarcopenia; 54 patients, 19.6%), osteo-frailty (patients with osteoporosis and frailty; 44 patients, 15.9%), and osteo-sarco-frailty (patients with osteoporosis, sarcopenia, and frailty; 14 patients, 5.1%) among patients with osteoporosis. Additionally, Fig 3 shows the prevalence of non-frail, pre-frail, and frail patients with OP and OS according to age category. The presence of osteo-sarcopenia increased in an age-dependent fashion (9.5%, 22.2%, and 29.5% in patients aged 65–74, 75–84, and ≥85 years, respectively). The population of patients who were frail or pre-frail was greater in OS than in OP, except in patients aged 65–74 years (65–74 years: 76.9% vs. 80.4%; 75–84 years: 85.7% vs. 79.8%; ≥85 years: 100% vs. 77.4%). The association of OS with frailty, assessed by logistic analysis using OP as the reference, is shown in Table 1. The likelihood of being frail was higher in the OS than in the OP (odds ratio [OR] = 2.33, 95% confidence interval [CI] 1.13–4.80; P = 0.028).

**Table 1:** Association between OS and frailty, with OP as the reference.

		OR (95% CI)	P value
All patients (N = 276)	OP	-	-
	OS	2.33 (1.13–4.80)	0.028*

* P < 0.05.

**Abbreviations**: OR, odds ratio; CI, confidence interval; OP, osteoporosis alone group; OS, osteosarcopenia group.

#### Comparison of clinical parameters between osteoporosis alone and osteosarcopenia groups

Comparative data on the clinical parameters are provided in Table 2. There were significant differences between OP and OS in age, BMI, Fried frailty score, serum IGF-1 levels, FN-BMD, RUD-BMD, SMI, grip strength, walking speed, bisphosphonate treatment, and selective estrogen receptor modulator (SERM) treatment. There were no significant differences in the other parameters. This pattern of results was almost replicated within each age category. However, in patients aged 65–74 years, but not in other age categories, there were significant differences between OS and OP in eGFR (72.00 (62.50–81.00) vs. 55.00 (50.80–68.50) mL/min/1.73 m^2^, P < 0.01), and HbA1c (5.79 ± 0.50% vs. 6.24 ± 0.95%, P = 0.04).

**Table 2:** Comparison of clinical parameters between patients with osteoporosis alone and patients with osteosarcopenia, overall and according to age category.

	All patients (≥65 years)				65–74 years				75–84 years				≥85 years			
All (N = 276)	OP (N = 222)	OS (N = 54)	P value	All (N = 105)	OP (N = 92)	OS (N = 13)	P value	All (N = 126)	OP (N = 99)	OS (N = 28)	P value	All (N = 44)	OP (N = 31)	OS (N = 13)	P value
Age (years)	77.07 ± 6.74	76.64 ± 6.81	78.83 ± 6.22	0.02*	70.21 ± 2.68	70.15 ± 2.66	70.62 ± 2.87	0.65	78.09 ± 2.95	79.13 ± 2.97	78.96 ± 2893	0.72	87.59 ± 2.77	87.94 ± 3.09	86.77 ± 1.64	0.40
Height (cm)	150.05 (145.75–154.50)	150.20 (146.00–154.60)	148.90 (145.40–153.70)	0.25	153.70 (149.28–156.40)	153.75 (149.40–156.45)	152.90 (148.80–155.85)	0.80	149.80 (145.30–154.10)	149.80 (145.85–153.28)	149.20 (145.30–154.10)	0.93	144.25 (140.45–148.20)	144.00 (138.38–147.73)	146.40 (142.83–148.90)	0.26
Weight (kg)	44.00 (42.60–54.20)	50.45 (45.10–55.45)	41.15 (37.90–44.90)	<0.01**	50.10 (44.63–55.46)	51.15 (45.13–56.75)	42.90 (40.90–47.93)	<0.01**	48.80 (41.93–53.50)	50.70 (46.04–54.98)	40.98 (38.85–42.65)	<0.01**	46.20 (40.58–51.08)	48.05 (43.40–52.89)	38.85 (36.40–45.13)	<0.01**
BMI (kg/m^2^)	21.90 (19.30–24.10)	22.65 (20.50–24.60)	18.55 (17.00–20.50)	<0.01**	21.70 (19.68–23.60)	22.10 (19.95–23.90)	20.20 (17.23–20.65)	<0.01**	21.80 (19.30–24.33)	22.90 (21.10–24.78)	18.30 (17.05–19.20)	<0.01**	22.60 (19.25–24.80)	23.40 (21.30–25.15)	18.20 (16.58–22.08)	<0.01**
Obesity [N (%)]	10 (3.6)	10 (4.5)	0 (0)	0.11	4 (3.8)	4 (43.5)	0 (0)	0.44	2 (1.6)	2 (2.0)	0 (0)	0.45	4 (9.1)	4 (12.9)	0 (0)	0.17
Overweight [N (%)]	94 (34.1)	92 (41.4)	2 (3.7)	<0.01**	30 (28.6)	30 (32.6)	0 (0)	0.01*	47 (34.6)	47 (47.5)	0 (0)	<0.01**	17 (38.6)	15 (48.4)	2 (15.4)	0.04*
Normal weight [N (%)]	130 (47.1)	103 (46.4)	27 (50.0)	0.85	56 (53.3)	47 (51.1)	9 (69.2)	0.22	59 (46.8)	45 (45.5)	14 (50.0)	0.67	15 (34.1)	11 (35.5)	4 (30.8)	0.76
Emaciation [N (%)]	42 (15.2)	17 (7.7)	25 (46.3)	<0.01**	15 (4.8)	11 (12.0)	4 (30.8)	0.07	19 (15.1)	5 (5.1)	14 (50.0)	<0.01**	8 (18.2)	1 (3.2)	7 (53.8)	<0.01**
Fracture history [N (%)]	149 (54.0)	120 (54.1)	29 (53.7)	0.96	43 (41.0)	35 (38.0)	8 (61.5)	0.11	79 (62.7)	61 (61.6)	18 (64.3)	0.84	35 (79.5)	27 (87.1)	8 (61.5)	0.06
Fall risk score	4.47 ± 3.28	5.26 ± 3.38	4.28 ± 3.23	0.05	3.00 ± 2.80	2.99 ± 2.76	3.08 ± 3.20	0.93	4.90 ± 3.20	4.69 ± 3.14	5.64 ± 3.36	0.19	6.77 ± 2.92	6.84 ± 3.05	6.62 ± 2.72	0.69
Fried frailty score	1.00 (1.00–2.00)	1.00 (1.00–2.00)	2.00 (1.00–3.00)	<0.01**	1.00 (1.00–2.00)	1.00 (1.00–2.00)	1.00 (0.80–2.00)	0.77	2.00 (1.00–2.00)	1.00 (1.00–2.00)	2.00 (1.50–2.50)	0.02*	2.00 (1.00–3.00)	2.00 (1.00–2.80)	2.00 (1.00–3.00)	0.25
**Laboratory Data**																
Serum Alb (g/dL)	4.18 ± 0.33	4.18 ± 0.33	4.19 ± 0.35	0.64	4.26 ± 0.32	4.26 ± 0.32	4.26 ± 0.33	0.75	4.17 ± 0.28	4.16 ± 0.26	4.20 ± 0.36	0.21	4.03 ± 0.42	4.02 ± 0.46	4.07 ± 0.35	0.91
Serum ALP (U/L)	199.95 ± 66.79	201.00 ± 65.25	195.58 ± 73.35	0.43	202.34 ± 62.38	205.52 ± 63.55	178.25 ± 48.23	0.39	197.24 ± 70.48	196.20 ± 68.98	200.93 ± 76.77	0.87	202.18 ± 67.00	203.06 ± 58.46	200.08 ± 86.79	0.53
Serum Ca (mg/dL)	9.51 ± 0.44	9.51 ± 0.42	9.50 ± 0.52	0.64	9.49 ± 0.47	9.51 ± 0.41	9.37 ± 0.78	0.91	9.49 ± 0.41	9.47 ± 0.41	9.55 ± 0.41	0.37	9.60 ± 0.46	9.64 ± 0.47	9.52 ± 0.45	0.51
Serum P (mg/dL)	3.74 ± 0.49	3.73 ± 0.48	3.79 ± 0.56	0.39	3.81 ± 0.54	3.80 ± 0.52	3.87 ± 0.68	0.80	3.71 ± 0.43	3.69 ± 0.41	3.76 ± 0.51	0.42	3.69 ± 0.55	3.66 ± 0.56	3.78 ± 0.55	0.49
Serum intact-PTH (pg/mL)	37.13 ± 27.19	36.45 ± 21.11	39.94 ± 44.36	0.51	35.47 ± 16.20	35.69 ± 16.64	33.75 ± 12.76	0.90	36.23 ± 22.18	36.33 ± 21.85	35.86 ± 23.71	0.40	33.93 ± 17.10	34.71 ± 17.10	32.08 ± 8.55	0.89
Serum IGF-1 (ng/mL)	90.18 ± 32.30	93.64 ± 33.01	75.94± 24.80	<0.01**	104.41 ± 31.38	106.92 ± 31.52	86.62± 24.63	0.03*	85.35 ± 30.37	88.12 ± 31.13	75.54 ± 25.67	0.15	70.16 ± 24.84	71.84 ± 26.74	66.15 ± 19.98	0.96
Serum 25-(OH) D (ng/mL)	19.00 (14.80–23.65)	18.60 (14.85–23.18)	20.30 (13.00–25.30)	0.43	18.70 (15.33–24.13)	18.30 (15.15–23.55)	23.90 (17.40–26.50)	0.10	19.40 (15.53–24.03)	19.40 (15.93–23.80)	19.10 (12.90–24.70)	0.69	16.75 (12.10–21.40)	16.10 (11.90–20.58)	19.20 (13.30–22.10)	0.31
eGFR (mL/min/1.73 m^2^)	63.00 (54.00–75.00)	64.00 (55.00–75.00)	60.00 (51.00–74.00)	0.32	70.00 (59.00–81.00)	72.00 (62.50–81.00)	55.00 (50.80–68.50)	<0.01**	61.00 (53.00–72.80)	61.00 (53.00–71.00)	64.00 (53.50–76.50)	0.32	55.50 (42.50–65.50)	54.50 (42.00–61.80)	59.0 (49.80–69.80)	0.19
HbA1c (%)	5.87 ± 0.70	5.84 ± 0.69	5.95 ± 0.71	0.56	5.85 ± 0.59	5.79 ± 0.50	6.24 ± 0.95	0.04*	5.90 ± 0.80	5.89 ± 0.85	5.92 ± 0.60	0.86	5.81 ± 0.63	5.86 ± 0.65	5.72 ± 0.60	0.35
**Bone Turnover Markers**																
Serum BAP (μg/L)	9.85 ± 4.72	9.80 ± 3.79	10.06 ± 7.47	0.43	9.75 ± 3.57	9.91 ± 3.63	8.58 ± 2.96	0.33	9.85 ± 4.30	9.85 ± 4.10	9.85 ± 5.06	0.73	9.47 ± 3.77	9.32 ± 3.29	9.83 ± 4.87	0.86
Urinary NTX (nmol∙BCE/mmol∙CRE)	27.38 ± 19.03	27.38 ± 19.45	25.28 ± 17.23	0.14	26.80 ± 16.02	28.06 ± 16.23	17.25 ± 9.69	0.03*	27.32 ± 18.52	27.74 ± 18.79	25.85 ± 17.78	0.38	27.48± 15.26	25.81 ± 13.07	31.46 ± 19.56	0.40
**DXA Measurement**																
LS-BMD (g/cm^2^)	0.78 (0.67–0.87)	0.79 (0.68–0.89)	0.74 (0.63–0.84)	0.06	0.79 (0.67–0.87)	0.80 (0.69–0.88)	0.74 (0.61–0.85)	0.20	0.79 (0.66–0.86)	0.79 (0.66–0.87)	0.77 (0.64–0.84)	0.60	0.76 (0.70–0.91)	0.78 (0.73–0.99)	0.74 (0.64–0.84)	0.05
FN-BMD (g/cm^2^)	0.51 (0.47–0.56)	0.52 (0.48–0.57)	0.48 (0.41–0.55)	<0.01**	0.52 (0.48–0.57)	0.53 (0.49–0.57)	0.43 (0.40–0.57)	0.09	0.52 (0.47–0.56)	0.53 (0.48–0.58)	0.49 (0.40–0.54)	0.02*	0.48 (0.43–0.55)	0.48 (0.43–0.55)	0.47 (0.43–0.50)	0.50
RUD-BMD (g/cm^2^)	0.27 (0.23–0.30)	0.28 (0.24–0.30)	0.24 (0.21–0.26)	<0.01**	0.29 (0.26–0.32)	0.29 (0.27–0.32)	0.25 (0.26–0.32)	0.14	0.26 (0.23–0.29)	0.27 (0.24–0.29)	0.23 (0.21–0.25)	<0.01**	0.24 (0.21–0.28)	0.24 (0.22–0.29)	0.24 (0.20–0.25)	0.22
SM**I** (kg/m^2^)	5.78 (5.30–6.30)	5.97 (5.60–6.41)	5.08 (4.73–5.30)	<0.01**	5.66 (5.28–6.22)	5.79 (5.43–6.25)	4.93 (4.76–5.29)	<0.01**	5.85 (5.31–6.32)	6.00 (5.73–6.45)	5.16 (4.82–5.30)	<0.01**	5.93 (5.23–6.59)	6.30 (5.72–6.98)	5.07 (4.51–5.20)	<0.01**
**Physical Performance Measurements**																
Grip strength (kg)	16.11 ± 5.01	17.04 ± 4.95	12.27 ± 3.01	<0.01**	18.57 ± 4.58	19.27 ± 4.30	13.61 ± 3.56	<0.01**	15.17 ± 4.56	16.06 ± 4.59	12.05 ± 2.78	<0.01**	12.93 ± 4.57	13.58 ± 5.02	11.39 ± 2.90	0.19
Walking speed (m/s)	1.00 ± 0.30	1.02 ± 0.31	0.91 ± 0.26	<0.01**	1.16 ± 0.26	1.17 ± 0.26	1.09 ± 0.24	0.10	0.96 ± 0.26	0.98 ± 0.26	0.90 ± 0.24	0.22	0.73 ± 0.26	0.70 ± 0.28	0.78 ± 0.20	0.19
**Drugs for Osteoporosis**																
None [N (%)]	17 (6.2)	14 (6.3)	3 (5.6)	0.84	6 (5.7)	6 (6.5)	0 (0)	0.34	8 (6.3)	6 (6.1)	2 (7.1)	0.84	3 (6.8)	2 (6.5)	1 (7.7)	0.88
Bisphosphonate [N (%)]	128 (46.4)	95 (42.8)	33 (61.1)	0.02*	51 (48.6)	42 (45.7)	9 (69.2)	0.11	49 (38.9)	36 (36.4)	13 (46.4)	0.33	28 (63.6)	17 (54.8)	11 (84.6)	0.06
SERM [N (%)]	59 (21.4)	54 (24.3)	5 (9.3)	0.02*	29 (27.6)	28 (30.4)	1 (7.7)	0.09	26 (20.6)	22 (22.2)	4 (14.3)	0.36	4 (9.1)	4 (12.9)	0 (0)	0.17
Denosumab [N (%)]	55 (19.9)	43 (19.4)	12 (22.2)	0.64	12 (11.4)	9 (9.8)	3 (23.1)	0.16	37 (29.4)	28 (28.3)	9 (32.1)	0.61	6 (13.6)	6 (19.4)	0 (0)	0.09
Teriparatide [N (%)]	5 (1.8)	5 (2.3)	0 (0)	0.27	3 (2.9)	3 (3.3)	0 (0)	0.51	2 (1.6)	2 (2.0)	0 (0)	0.45	0 (0)	0 (0)	0 (0)	1.00
Vitamin D [N (%)]	12 (4.3)	11 (5.0)	1 (1.9)	0.32	4 (3.8)	4 (4.3)	0 (0)	0.46	5 (4.0)	5 (5.1)	0 (0)	0.23	3 (6.8)	2 (6.5)	1 (7.7)	0.88

Data shown as n (%) were analyzed by χ2 test. Data expressed as mean ± SD and median (IQR: Q1–Q3) were analyzed by the Mann–Whitney U test.

* P < 0.05

** P < 0.01. BMI categories was defined as follows; Emaciation: less than 18.5 kg/m^2^, Normal weight: 18.5–22.9 kg/m^2^, Overweight: 23.0–27.4 kg/m^2^, Obesity: 27.5 kg/m^2^ or more.

**Abbreviations**: OP, osteoporosis alone group; OS, osteosarcopenia group; BMI, body mass index; Alb, albumin; ALP, alkaline phosphatase; Ca, calcium; P, phosphorus; intact-PTH, intact-parathyroid hormone; IGF-1, insulin-like growth factor 1; 25-(OH) D, 25-hydroxyvitamin D; eGFR, estimated glomerular filtration rate; HbA1c, hemoglobin A1c; BAP, bone-specific alkaline phosphatase; NTX, N-telopeptide of type I collagen; DXA, dual-energy X-ray absorptiometry; LS-BMD, bone mineral density of lumber spine; FN-BMD, bone mineral density of femoral neck; RUD-BMD, bone mineral density of the distal radius; SMI, skeletal muscle mass index; SERM, selective estrogen receptor modulator.

### Multivariate logistic regression analysis for the risk factors of osteosarcopenia

The results of the multivariate logistic regression analysis are shown in Table 3. Although BMI and serum IGF-1 levels were identified as independent predictors of osteosarcopenia among all patients, BMI was the only remaining factor after adjustment for age (BMI: OR = 1.71, 95% CI: 1.46–2.00, P < 0.01). This pattern of results was similarly obtained within the age categories of 75–84 and ≥85 years. However, in patients aged 65–74 years, BMI, eGFR, and HbA1c levels were identified as independent predictors of osteosarcopenia, and interestingly, all of these factors remained after adjustment for age (BMI: OR = 1.69, 95% CI: 1.22–2.32, P < 0.01; eGFR: OR = 1.75, 95% CI: 1.12–2.72, P = 0.01; and HbA1c: OR = 5.01, 95% CI: 1.40–18.55, P = 0.01).

**Table 3:** Multivariate logistic regression analysis for the risk factors of osteosarcopenia, overall and according to age category.

	Factors	OR (95% CI)	P value	OR^a^ (95% CI)	P^a^ value
**All**	BMI (per 1 kg/m^2^ decrease)	1.70 (1.45–1.99)	<0.01**	1.71 (1.46–2.00)	<0.01**
	Fall risk score (per 1point increase)	1.11 (0.99–1.25)	0.05	1.01 (0.97–1.24)	0.14
	Serum IGF-1 levels (per 10 ng/mL increase)	1.20 (1.05–1.37)	<0.01**	1.16 (0.99–1.35)	0.06
**65–74 years**	BMI (per 1 kg/m^2^ decrease)	1.67 (1.21–2.31)	<0.01**	1.69 (1.22–2.32)	<0.01**
	eGFR (per 10 mL/min/1.73 m^2^ decrease)	1.71 (1.11–2.65)	0.02*	1.75 (1.12–2.72)	0.01*
	serum IGF-1 levels (per 10 ng/mL increase)	1.17 (0.89–1.54)	0.25	1.15 (0.87–1.52)	0.32
	HbA1c (per 1% decrease)	5.13 (1.38–19.07)	0.01*	5.10 (1.40–18.55)	0.01*
**75–84 years**	BMI (per 1 kg/m^2^ decrease)	2.26 (1.66–3.19)	<0.01**	2.30 (1.66–3.19)	<0.01**
**≥85 years**	BMI (per 1 kg/m^2^ decrease)	1.53 (1.16–2.03)	<0.01**	1.52 (1.15–2.02)	<0.01**

* P < 0.05

** P < 0.01

^a^ Age-adjusted model.

**Abbreviations**: OR, odds ratio; CI, confidence interval; BMI, body mass index; IGF-1, insulin-like growth factor 1; eGFR, estimated glomerular filtration rate; HbA1c, hemoglobin A1c; NTX, N-telopeptide of type I collagen; RUD-BMD, bone mineral density of the distal radius.

## Discussion

Osteosarcopenia contributes to a high risk of falls, fracture, institutionalization, depression, and poor quality of life [25, 26]. Furthermore, osteosarcopenia is associated with substantially increased morbidity and mortality, which places a significant financial burden on the healthcare system [9]. Yoshimura et al., in a 4-year follow-up survey, reported that the presence of osteoporosis significantly increases the future risk of developing osteosarcopenia [14]. However, exercise and nutrition interventions for the treatment of sarcopenia may play a role in improving muscle strength [15]. Hence, early detection of sarcopenia development in patients with osteoporosis might be helpful in preventing negative outcomes. The present study is the first to report that patients with osteoporosis aged 65–74 years who have comorbidities such as kidney dysfunction and abnormal glucose tolerance are at an increased risk of developing osteosarcopenia. Moreover, low BMI was the strongest risk factor for all age categories. Although the study results may be relatively intuitive, they have not been previously established in this high-risk population.

In the present study, we used the JOS-criteria and AWGS consensus to define osteosarcopenia [17, 24]. The cut-off values for physical performance in Asian populations differ from those in Caucasian populations because of differences in ethnicity, body size, lifestyle, and cultural backgrounds [17, 27, 28]. The prevalence of osteosarcopenia (19.6%) was higher in the present study than in previous studies [10, 11, 14]. Specifically, a population-based cohort study conducted in Japan reported that 6.2% of women aged ≥60 years had osteosarcopenia [10, 11, 14]. Additionally, Wang et al. reported that the prevalence of osteosarcopenia in community-dwelling, older Chinese women aged >65 years was 15.1% [10]. The greater prevalence of osteosarcopenia in the present study may be due to our focus on a high-risk population.

The present study results suggest that the components of the diagnosis of sarcopenia assessed in an osteoporosis center have relevance. To identify the risk factors of osteosarcopenia development, we compared various clinical parameters between OP and OS. Although there was a significant age difference between OP and OS, some parameters showed a significant difference, indicating that the risk of osteosarcopenia development may be detected. Thus, we further analyzed the patients according to age category (65–74, 75–84, and ≥85 years) to minimize the effects of bias. We first investigated the prevalence of frailty within each age category to elucidate the magnitude of the impact of osteosarcopenia on daily life. A recent study showed that prevention of osteoporosis and osteosarcopenia may help in reducing the risk of frailty [11]. Furthermore, a previous study conducted in China reported that the likelihood of frailty was substantially higher in the presence of osteosarcopenia in the female population (OR 4.67; 95% CI, 2.42–18.86; P = 0.007) [10]. Another study conducted in Japan showed that the risk of frailty significantly increased with the presence of osteosarcopenia (OR 5.80; 95% CI, 1.38–24.4; P = 0.017) [11]. Consistent with these findings, we found that among high-risk patients, those with osteosarcopenia were more likely to be frail than were patients with osteoporosis alone. As frailty has a strong connection with independent living, these results support our concept that there is a possibility of reducing the risk of frailty if we can prevent the development of osteosarcopenia in patients with osteoporosis.

We also compared various baseline clinical demographic parameters between OP and OS within each age category. We found that patients with osteosarcopenia have a significantly lower BMI than do patients with osteoporosis alone. Moreover, none of the patients with osteo-sarcopenia were obese (BMI ≥27.5 kg/m^2^). BMI remained unaltered following multiple regression analysis within all age categories and seemed to be the strongest factor associated with the development of osteosarcopenia. High and low BMIs are known risk factors of osteoporosis [29, 30]. However, to the best of our knowledge, the association between low BMI and sarcopenia has received less attention compared to that for high BMI and sarcopenia (known as sarcopenic obesity) [14, 31, 32]. The findings of the present study are supported by those of a recent study that reported emaciation as being significantly associated with both osteoporosis and sarcopenia [14]. The authors claimed that high BMI might not be associated with the existence of sarcopenia and suggested that the definition of sarcopenic obesity should incorporate severe health illnesses such as cardiovascular disease [14]. Indeed, sarcopenia and cardiac hypertrophy are not rare in the geriatric population, and the evidence suggests that sarcopenia with obesity may be associated with higher levels of metabolic disorders and an increased risk of mortality compared to that for obesity or sarcopenia alone [33].

The multivariate logistic analysis revealed that the risk factors of developing osteosarcopenia were different across age categories. Notably, factors related to comorbidities, such as HbA1c and eGFR, were detected as risk factors of osteosarcopenia among patients aged 65–74 years. These findings are supported by those of a previous study that showed that a higher level of comorbidity (the presence of more than three of the chronic diseases) was associated with osteosarcopenia, even though they assessed the presence of comorbidities by a physician’s diagnosis [10]. Furthermore, Tanaka et al showed that advanced glycation end-products (AGEs) suppressed the expression of myogenic genes, such as MyoD and myogenin, as well as osteoglycin in myoblasts [34]. In patients with high levels of HbA1c, AGEs may be involved in muscle wasting and impairment of osteoglycin-mediated interactions between muscle and bone [34]. Overall, obtaining appropriate assessments of the presence of comorbidities, including HbA1c and kidney function, using blood samples may help in identifying patients that are at a greater risk of developing osteosarcopenia.

Endocrine disorders such as diabetes, thyroid function disorder, low levels of Vitamin D, sex, steroid abnormalities, reduced growth hormone/IGF-1, and malnutrition have also been associated with osteosarcopenia [9, 17, 35–38]. In the present study, low IGF-1 was an insignificant independent risk factor after adjustment for age because a negative correlation was observed between age and IGF-1 levels (r = −0.42, P < 0.01, S1 Fig) Other factors related to endocrine disorders were not identified as risk factors of osteosarcopenia development, with the exception of high HbA1c. These results may be because we evaluated only patients with osteoporosis. Additionally, osteoporosis and sarcopenia are multifactorial and share various pathophysiological pathways [12].

The present study had several limitations. First, the number of patients and risk factors were inadequate for drawing conclusions regarding the risk of osteosarcopenia because of the retrospective cross-sectional study design. The participants may not be representative of the high-risk population. Moreover, we did not assess patients who were bedridden, which represents the end stage of frailty, as we only analyzed patients with independence. Thus, future prospective analyses with a larger number of patients, including those with impaired activities of daily living, are warranted.

Second, in the present study, walking test was measured only once. We might not exclude the effect of incidental changes in participants’ performance. To minimize fluctuation of measurements, more recurrent analysis should be taken into consideration [39]. Third, we did not assess drug effects except for that of glucocorticoid. Many types of drugs have been shown to be associated with osteoporosis and fragility fractures in epidemiological studies [40] with the possibility of an association to sarcopenia. Finally, we could not analyze the risk factors of sarcopenia development between patients with osteoporosis and healthy controls, and it remains unclear which condition developed first, sarcopenia and osteoporosis. However, patients with osteoporosis are at a greater risk of osteosarcopenia development.

## Conclusions

The present study is the first to demonstrate the risk factors of developing osteosarcopenia, as assessed in an osteoporosis clinic. The results suggest that patients with low BMI have a higher risk of developing osteosarcopenia. Moreover, appropriate assessments, including the presence of comorbidities, will help in identifying patients at greater risk of developing osteosarcopenia, especially for patients aged 65–74 years. Further studies with long-term prospective results are needed to validate our study findings.

## Supporting information

S1 Table A simple screening test for the risk of falls in community-dwelling elder persons. (DOCX)

S1 Fig Correlation of the serum IGF-1 levels and Age in all patients. **Abbreviations**: IGF-1, insulin-like growth factor 1.
(TIF)
