# Supplementary material for: Risk factors predicting osteosarcopenia in postmenopausal women with osteoporosis: A retrospective study
Source: PLoS One. 2020 Aug 7;15(8):e0237454. doi: 10.1371/journal.pone.0237454 (PMC7413553; doi:10.1371/journal.pone.0237454)
Supplement: S1 Table — (DOCX) [file pone.0237454.s001.docx]

**S1 Table.**: A simple screening test for the risk of falls in community-dwelling elder persons

| **Questionnaire items** | **point** |
| --- | --- |
| History of fall within one year | Yes=5, No=0 |
| Do you feel your walking speed declined recently? | Yes=2, No=0 |
| Do you use cane when you walk? | Yes=2, No=0 |
| Is your back bended? | Yes=2, No=0 |
| Do you take more than five kinds of prescribed medicines? | Yes=2, No=0 |

**Note**: Cut-off values is > 6 points [Odds ratio of 3.88 (95% CI: 3.16–3.88)].
