# Supplementary figures and images for: Risk factors predicting osteosarcopenia in postmenopausal women with osteoporosis: A retrospective study
Source: PLoS One. 2020 Aug 7;15(8):e0237454. doi: 10.1371/journal.pone.0237454 (PMC7413553; doi:10.1371/journal.pone.0237454)

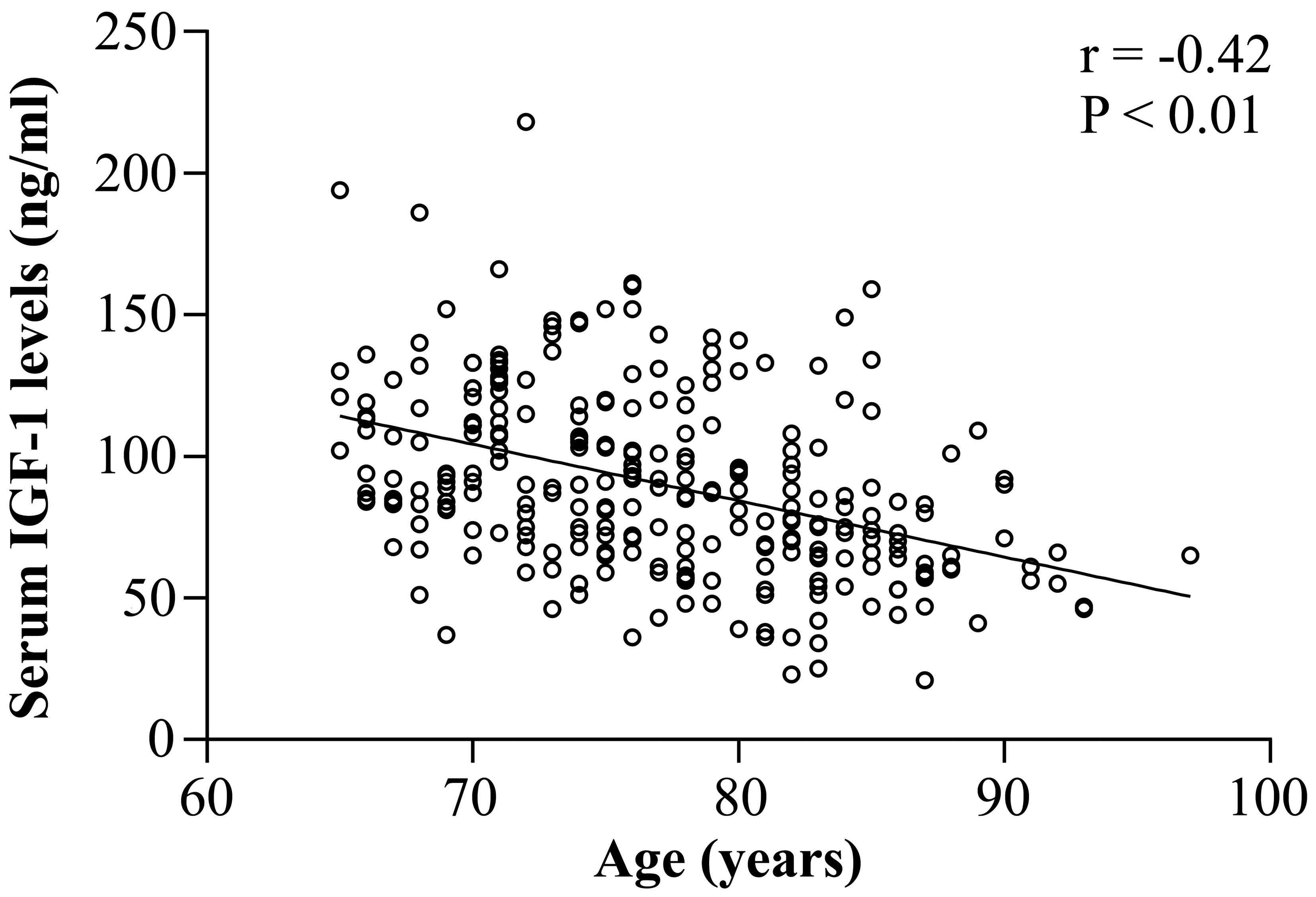

Supplement: S1 Fig — Abbreviations: IGF-1, insulin-like growth factor 1. (TIF) [file pone.0237454.s002.tif]
